# Enhanced virulence of *Plasmodium falciparum* in blood of diabetic patients

**DOI:** 10.1371/journal.pone.0249666

**Published:** 2021-06-17

**Authors:** Jun-Hong Ch’ng, Kirsten Moll, Katja Wyss, Ulf Hammar, Mikael Rydén, Olle Kämpe, Anna Färnert, Mats Wahlgren

**Affiliations:** 1 Department of Microbiology, Tumor and Cell Biology (MTC), Karolinska Institutet, Stockholm, Sweden; 2 Department of Microbiology and Immunology, National University of Singapore, Singapore, Singapore; 3 Department of Medicine Solna, Karolinska Institutet, Stockholm, Sweden; 4 Department of Infectious Diseases, Karolinska University Hospital, Stockholm, Sweden; 5 Department of Epidemiology, Institute for Environmental Medicine, Karolinska Institutet, Stockholm, Sweden; 6 Department of Medicine Huddinge, Stockholm, Sweden; 7 Department of Endocrinology, Karolinska University Hospital, Stockholm, Sweden; Ehime Daigaku, JAPAN

## Abstract

Rising prevalence of diabetes in sub-Saharan Africa, coupled with continued malaria transmission, has resulted more patients dealing with both communicable and non-communicable diseases. We previously reported that travelers with type 2 diabetes mellitus (T2DM) infected with *Plasmodium falciparum* were three times more likely to develop severe malaria than non-diabetics. Here we explore the biological basis for this by testing blood from uninfected subjects with type 1 and type 2 diabetes, ex vivo, for their effects on parasite growth and rosetting (binding of infected erythrocytes to uninfected erythrocytes). Rosetting was associated with type 2 diabetes, blood glucose and erythrocyte sedimentation rate (ESR), while parasite growth was positively associated with blood glucose, glycated hemoglobin (HbA1c), body mass index (BMI), fibrinogen and triglycerides. This study establishes a link between diabetes and malaria virulence assays, potentially explaining the protective effect of good glycemic control against severe malaria in subjects with diabetes.

## Introduction

Diabetes affects an estimated 422 million people and is a major cause of morbidity such as kidney dysfunction, lower limb amputation, cardiovascular disease and blindness [1]. WHO estimated 3.7 million diabetes-related deaths in 2012 and projected the disease to be the seventh leading cause of death by 2030 [1]. Historically a disease associated with the wealthy, the prevalence of type 2 diabetes has been increasing rapidly in developing countries which also bear a high burden of communicable diseases such as HIV, tuberculosis and malaria [2]. In particular, sub-Saharan Africa has the highest prevalence of undiagnosed diabetes with 60% of diabetics being unaware they have the disease [3].

Some studies have investigated the correlations between diabetes and HIV infection [4, 5] and between diabetes and tuberculosis [6], but very few have considered the effects of diabetes on malaria morbidity and mortality. One study reported a higher prevalence of asymptomatic *Plasmodium falciparum* infection among type 2 diabetics in Ghana [7], and we recently showed that travelers diagnosed with *P*. *falciparum* in Sweden were three times more likely to develop severe malaria if they had T2DM [8]. One possible explanation for this association involves the atypical properties of red blood cells (RBCs) in diabetic patients which promote their aggregation [9, 10].

The etiology of diabetes and malaria could not be more different yet pathological complications arising from both diseases are attributed, at least in part, to altered RBC properties and reduced tissue perfusion. In diabetes, RBCs spontaneously and reversibly aggregate into stacks called rouleaux which reduces their flow through narrow capillaries [11–17]. Rouleaux tend to sink faster than normal RBCs, leading to an increased erythrocyte sedimentation rate (ESR), a commonly assayed hematological parameter. Rouleaux formation is attributed to the level of fibrinogen (and also other acute phase reactants) in plasma, as well as the reduction in negatively charges on the RBC surface (reduced cell-cell repulsive force called zeta potential) [10, 16, 18–21].

In malaria, the aggregation of uninfected RBCs around parasitized RBCs forms cell clusters called rosettes [22]. Rosettes become lodged in microvasculature and have been associated with malaria complications such as cerebral malaria and renal dysfunction [23, 24]. In contrast to the interactions during rouleaux formation (the strength of in vitro interactions of rouleaux formed in dextrans range from about 1.4×10^−11^ N to 1.69×10^−10^ N [25]), the interactions holding a rosette together (about 4.4×10^−10^ N [26]) is mediated by parasite proteins exported to the surface of the infected RBC such as PfEMP1, RIFINS and/or STEVORs [27]. These ligands recognize receptors on the uninfected erythrocytes including ABO blood group antigens and heparan sulfate [28–30], as well as serum proteins such as albumin, IgM, IgG, fibrinogen and von Willebrand factor [31–34].

We hypothesized that RBCs of diabetic patients will more readily form rosettes when infected with *P*. *falciparum*. The contribution of other clinical and blood parameters influencing parasite rosetting and growth is also considered, in particular, the effect of blood glucose. Glucose has been shown to be an essential and limiting substrate for parasite replication in vitro [35] but it has yet to be determined whether this is the case in nutrient-replete serum. Here, blood samples from uninfected subjects with type 1 and type 2 diabetes were compared to non-diabetic controls (all living in a non-endemic country) to evaluate the effect of RBC and/or serum on *P*. *falciparum* growth and rosetting *ex vivo*.

## Results

### Patient characteristics

Altogether 25 diabetics and 25 non-diabetic controls participated over two separate occasions (identical experimental design). Study 1 consisted of nine controls and twelve type 1 diabetics while Study 2 included sixteen controls, three type 1 and ten type 2 diabetics (S1 Table). A summary of characteristics is presented in Tables 1 and S1. Altogether, the mean age was 43 yrs (SD = 13.5, range: 23–73 yrs) and there were more female participants (62%). Participants were predominantly of blood group A (42%) and blood group O (40%). Mean HbA1c was 33.6 mmol/mol for non-diabetic controls, 63.1 mmol/mol for type 1 subjects and 83.8 mmol/mol for type 2 individuals while plasma glucose (non-fasting, sampled on arrival for blood collection) was 5.2 mM, 11.1 mM and 11.8 mM respectively. The mean BMI was 24.76 kg/m^2^ for healthy controls, 25.26 kg/m^2^ for type 1 diabetics and 31.81 kg/m^2^ for type 2 diabetics (Table 1); the proportion of obese (BMI>30 kg/m^2^) was 12% among the healthy controls, 13% among the type 1 diabetics and 50% among the type 2 diabetics (Table 1). Duration of diabetes and type of complications as well as ongoing medication, country of birth, travel history to the tropics and previous malaria exposure were also documented (S1 Data).

**Table 1:** Characteristics of the study population.

Group	Study1+2			
Subgroup	All	Healthy	Type 1	Type 2
**Participants, n (%)**	50 (100)	25 (50)	15 (30)	10 (20)
**Sex (female), n (%)**	31 (62)	19 (76)	8 (53)	4 (40)
**Age (yrs), mean [SD)**	43.2 [13.5]	40.4 [12.5]	38.4 [12.1]	57.3 [8.6]
**BMI (kg/m**^**2**^**), mean [SD]**	26.32 [5.0]	24.76 [4.4]	25.26 [3.6]	31.81 [4.7]
**BMI classification**^**1**^**, n (%)**				
Underweight	1 (2)	1 (4)	0 (0)	0 (0)
Normal	23 (26)	15 (60)	8 (53)	0 (0)
Overweight	16 (32)	6 (24)	5 (33)	5 (50)
Obese	10 (20)	3 (12)	2 (13)	5 (50)
**Blood group, n (%)**				
O	20 (40)	10 (40)	7 (47)	3 (30)
A	21 (42)	11 (44)	6 (40)	4 (40)
B	5 (10)	2 (8)	2 (13)	1 (10)
AB	4 (8)	2 (8)	0 (0)	2 (20)
**Clinical chemistry, mean [SD]**				
HbA1c (mmol/mol)	52.5 [24.1]	33.6 [3.7]	63.1 [17.5]	83.8 [19.4]
Blood glucose (mM)	8.3 [4.5]	5.2 [1.0]	11.1 [4.4]	11.8 [4.7]
Hematocrit (x10^12^/L)	4.3 [0.6]	4.2 [0.3]	4.7 [0.8]	4.2 [0.5]
MCV (fL)	87.6 [5.6]	89.5 [4.1]	86.0 [6.7]	85.2 [5.7]
ESR (mm/hr)	14.5 [18.5]	9.8 [7.8]	14.1 [15.8]	26.6 [33.0]
Fibrinogen (g/L)^2^	3.0 [0.7]	2.8 [0.6]	3.00 [0.6]	3.5 [0.9]
C-react. protein (mg/L)	3.2 [4.4]	2.5 [4.13]	3.7 [5.5]	4.0 [2.9]
Apolipoprotein B (g/L)^2^	0.9 [0.3]	0.9 [0.2]	0.8 [0.3]	1.1 [0.4]
Triglycerides (mM)	1.7 [1.8]	1.2 [0.5]	1.2 [0.6]	3.8 [3.2]
Cholesterol (mM)	5.0 [1.0]	5.2 [0.9]	4.7 [1.0]	5.1 [1.4]
HDL-cholesterol (mM)	1.6 [0.6]	1.8 [0.4]	1.7 [0.7]	1.2 [0.2]
LDL cholesterol (mM)^2^	2.6 [0.9]	2.8 [0.8]	2.4 [0.9]	2.3 [0.8]
LDL/HDL	1.8 [0.8]	1.7 [0.7]	1.6 [0.8]	2.2 [1.1]

^1^ WHO BMI classification for adults BMI <18.5 (underweight), 18.5–25 (normal), 25–30 (overweight), >30 obese.

^2^ Missing patient parameters (see S1 Data): apolipoprotein B was not measured for all nine healthy controls in Study 1 and from one control in Study 2, fibrinogen levels for one type 1 diabetic in Study 2 was missing and LDL-cholesterol could not be measured in two type 2 diabetics in Study 2 due to high glycerol levels that interfered with measurements.

### Parasite rosetting

The rosetting rate was significantly higher when parasites were grown with RBCs from type 2 diabetics compared to healthy controls (RBC assay, Study 2: 25.59% vs 17.11%; *P* = 0.006, Table 2). For type 1 diabetics, rosetting rate also tended to be higher compared to healthy controls, however, the difference was less prominent (RBC assay Study 1: 20.98% vs 18.03%, *P* = 0.072; RBC assay Study 2: 22.63% vs 17.11%, *P* = 0.052, Table 2).

**Table 2:** Association of variables with rosetting rate in red blood cell (RBC) assay.

Assay	Variable	Study 1				Study 2			
		Rosetting mean % multiplets [SD]	Coef.	95% CI	*P*	Rosetting mean % multiplets [SD]	Coef.	95% CI	*P*
**RBC**	Non-diabetic	18.03 [2.74]	0			17.11 [5.76]	0		
	Diabetes type 1	20.98 [4.02]	2.95	-0.29;6.19	0.072	22.63 [3.19]	5.52	-0.04;11.09	0.052^2^
	Diabetes type 2					25.59 [6.76]^1^	8.48	2.68;14.28	0.006^3^
	Non-obese	20.20 [3.85]	0			20.05 [7.22]	0		
	Obese	16.84 [0.61]	-3.36	-5.51;-1.21	0.004	21.8 [6.43]	1.75	-4.99;8.49	0.598
	Male	18.58 [2.02]	0			20.34 [7.84]	0		
	Female	20.18 [4.23]	1.60	-1.43;4.63	0.284	20.49 [6.53]	0.16	-5.81;6.12	0.957
	Age		-0.07	-0.20;0.05	0.212		0.18	0.01;0.35	0.044
	BMI		-0.13	-0.65;0.39	0.614		0.36	-0.09;0.80	0.113
	Blood group O	22.90 [1.97]	0			17.47 [6.10]	0		
	Blood group A	16.84 [1.06]	-6.06	-7.82;-4.30	<0.001	22.70 [7.67]	5.23	-1.05;11.51	0.099
	Blood group B	22.83 [7.37]	-0.08	-15.7;15.54	0.992	21.11 [6.95]	3.64	-7.19;14.48	0.494
	Blood group AB	16.83 [0.33]	-6.07	-7.79;-4.35	<0.001	24.67 [4.67]	7.20	-3.15;17.55	0.164
	Blood glucose (mM)		4.59	1.31;7.88	0.009		7.07	1.94;12.20	0.009
	ESR (mm/Hr)		1.24	-0.03;2.50	0.056		2.31	0.34;4.28	0.023
	HbA1c (mmol/mol)		3.65	-1.50;8.80	0.154		9.16	3.65;14.67	0.002
	Fibrinogen (g/L)		1.80	-0.73;4.33	0.153		3.07	0.55;5.59	0.019
	Triglycerides (mM)		-0.35	-5.67;4.98	0.893		3.25	-0.80;7.29	0.111
	Cholesterol (mM)		-5.70	-14.61;3.21	0.197		3.22	-10.51;16.95	0.634

¹ RBCs from one type 2 diabetic (Donor 6.27 on 14 different of medications) showed atypical forward- and side-scatter profiles on the cytometer and accurate determination of the percentage of multiplets was not possible.

² Type II diabetics compared with non-diabetics.

^3^ Type I diabetics compared with non-diabetics.

Parasites grown in the serum from type 2 diabetics had slightly lower rosetting rates than the healthy controls (Serum assay, Study 2: 31.03% vs 33.99%, *P* = 0.015, S2 Table). However, the biological relevance of such a small reduction is uncertain but could potentially be due to serum components unique to this group or because of interference of ongoing medications (see S1 Data for list of medications). There was no difference in rosetting rate between parasites cultured in serum from type 1 diabetics or healthy controls (S2 Table).

Blood glucose correlated to rosetting rate in the RBC assay in both Study 1 and Study 2 (*P* = 0.009 for both studies, Table 2), however, no significant correlation was seen in the serum or the RBC+serum assay (S2 Table). HbA1c also correlated positively to rosetting rate in the RBC assay for Study 2 (*P* = 0.002, Table 2) and the RBC+serum assay for Study 1 (*P =* 0.021, S2 Table).

ESR correlated with rosetting rate in the RBC assay of Study 2 (*P* = 0.023) and was borderline in Study 1 (*P* = 0.056) (Table 2). This suggests that RBCs prone to rouleaux formation (and therefore sediment more readily) give rise to higher parasite rosetting rates.

Interestingly, fibrinogen also associated positively with rosetting rate in the RBC assay of Study 2 (*P =* 0.019, Table 2) despite these cells being washed (free of fibrinogen) suggesting that other cell-based factors related to fibrinogen (such as sialic acid levels [36]) could be involved.

**Fig 1:**
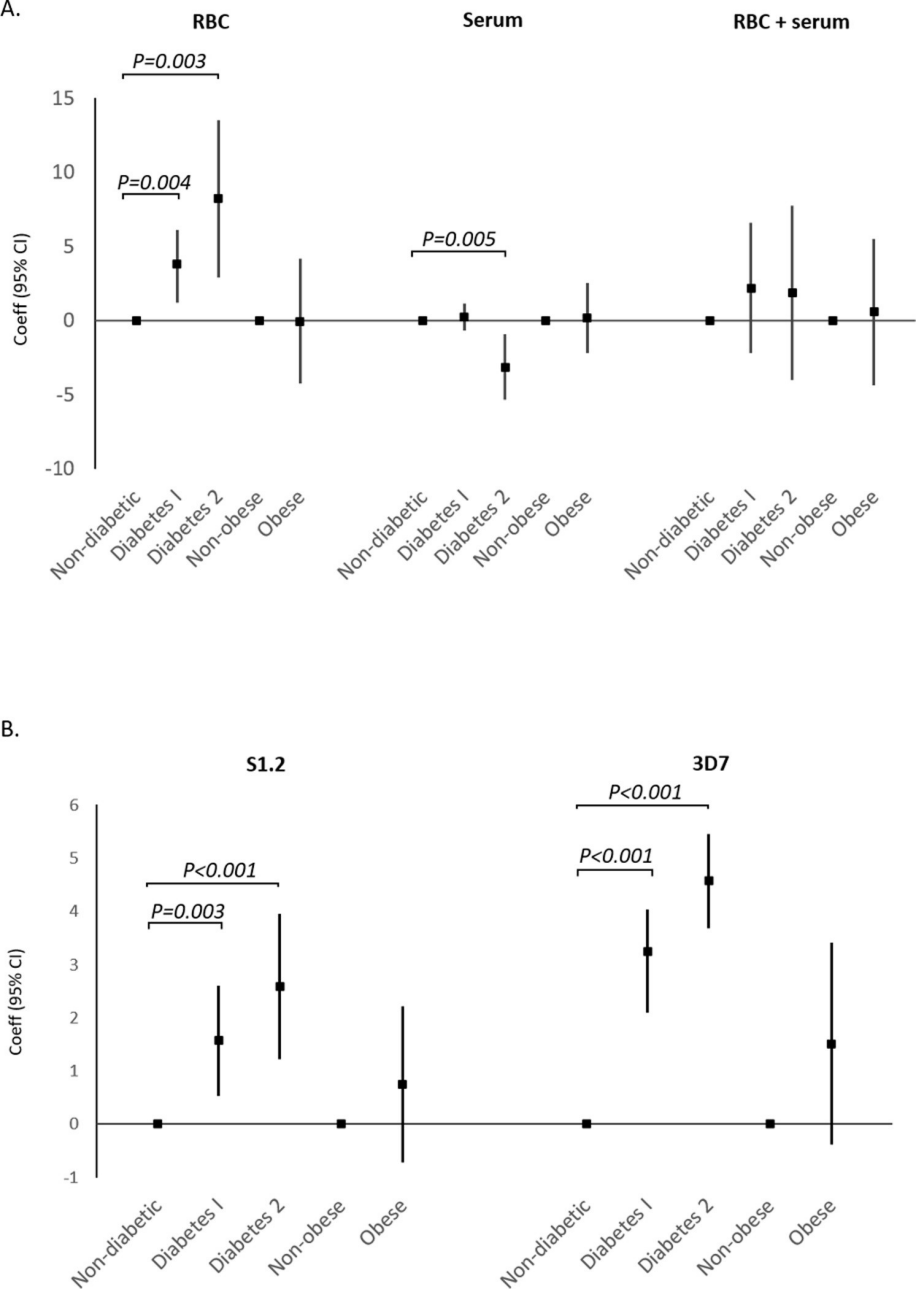


When merging the data sets (Study 1 + Study 2) in multivariate analyses, both diabetes type 1 and type 2 were associated with higher rosetting rate in the RBC assay with adjustment for Study (type 1 diabetes: *P* = 0.004, type 2 diabetes: *P* = 0.003, Fig 1A and S3 Table). After additional adjusting for age and BMI, diabetes type 1 and 2 was still significantly associated with rosetting (type 1 diabetes: *P* = 0.038, type 2 diabetes: *P* = 0.011, S3 Table). However, when also adjusting for glucose and ESR, only the association with type 2 diabetes was significant (type 1 diabetes: *P* = 0.076, type 2 diabetes: *P* = 0.020, S3 Table). There were no significant associations in the serum and RBC+serum assays except for type 2 diabetes, which again showed negative correlation to rosetting in serum assay even after adjustments (*P* = 0.001, S3 Table). There were no significant correlations between obesity and rosetting rate for any of the assays after pooling data from both studies (S3 Table).

There were some additional findings that were statistically significant but less robust due to limited power or the influence of confounders: A significantly lower rosetting rate was observed in the RBC assay of obese individuals compared to non-obese in Study 1 (*P* = 0.004) but not in Study 2 (*P* = 0.598) (Table 2). However, there were only 3 obese individuals in Study 1 and two of these were controls, as opposed to Study 2 where only one of the six obese was from the control group (S1 and S2 Tables). There was no correlation with sex but a small positive correlation with age was observed in RBC assay of Study 2 (*P* = 0.044, Table 2) and the serum assay of Study 1 (*P* = 0.037, S2 Table). Though there was a negative correlation for blood group AB (Study 1 RBC assay, *P*<0.001, Table 2; Study 2 RBC+serum assay, *P =* 0.034, S2 Table), this observation was based on only 2 individuals with blood group AB (both in healthy controls). For blood group A there was a significantly lower rosetting rate compared to blood group O in Study 1 of the RBC assay (16.84% vs 22.90%, *P*<0.001, Table 2). This was unexpected since S1.2 is known to form larger rosettes with blood group A and AB erythrocytes [29], which might explain why there was a tendency of higher rosetting rate for blood group A in Study 2 (22.70% vs 17.47%, *P* = 0.099). These contradictory findings could be due to the uneven blood group distributions in study groups (S1 Table). Rosetting rate also correlated to total cholesterol in the serum assay of Study 1 but not in Study 2(*P* = 0.001 and *P* = 0.138 respectively, S2 Table), and rosetting negatively correlated to triglycerides in Study 2 for both serum and RBC+serum assays (*P =* 0.002 and *P =* 0.049 respectively, S2 Table). The heterogenous results in the two studies prevent us from drawing any reliable conclusions and could be confounded by the diabetes status in the two groups (type 2 diabetics have higher triglycerides and cholesterols than type 1, Table 1).

### Parasite growth

In univariable analyses, serum from both type 1 and type 2 diabetics resulted in significantly higher parasite growth rates when compared to controls for both S1.2 (mean parasitemia fold-change 0.8 for non-diabetic controls, 2.39 for type 1 diabetics and 3.38 for type 2 diabetics. *P* = 0.001 and <0.001 respectively when compared to controls, Table 3) and 3D7 assay (mean parasitemia fold-change 1.94 for non-diabetic controls, 5.54 for type 1 diabetics and 6.18 for type 2 diabetics; *P*<0.001 when comparing both diabetic types to controls).

**Table 3:** Association of diabetes and obesity with parasite growth in multivariable analyses (Study 1+2).

Parasite strain	Variable	Parasite growth		Adjusted^1^			Adjusted^2^			Adjusted^3^		
		Mean^4^ [SD]	*P*	Coef.	95%CI	*P*	Coef.	95%CI	*P*	Coef.	95%CI	*P*
**S1.2**	Non-diabetic	0.80 [0.25]		0			0			0		
	Diabetes type 1	2.39 [1.65]	0.001	1.57	0.56;2.57	0.003	1.41	0.21;2.62	0.023	-0.94	-1.78;-0.09	0.031
	Diabetes type 2	3.38 [2.08]	<0.001	2.58	1.24;3.93	<0.001	2.69	1.09;4.28	0.002	-0.05	-1.59;1.49	0.948
	Non-obese	1.64 [1.47]		0						0		
	Obese	2.41 [2.22]	0.197	0.75	-0.69;2.19	0.301	0.65	-0.96;2.27	0.421	0.17	-0.60;0.93	0.663
**3D7**	Non-diabetic	1.94 [1.10]		0			0			0		
	Diabetes type 1	5.54 [1.77]	<0.001	3.24	2.12;4.36	<0.001	3.17	1.80;4.53	<0.001	1.12	0.45–2.20	0.042
	Diabetes type 2	6.18 [1.10]	<0.001	4.57	3.71;5.43	<0.001	4.08	2.73;5.43	<0.001	0.81	-0.85;2.47	0.327
	Non-obese	3.53 [2.31]		0			0			0		
	Obese	4.89 [2.32]	0.106	1.51	-0.36;3.38	0.111	1.21	-0.72;3.14	0.213	.07	-0.83;0.97	0.873

^1^ Adjusted for study.

^2^ For diabetes adjusted for study, age and BMI; for obesity adjusted for study & age.

^3^ For diabetes adjusted for study, age, BMI, blood glucose, cholesterol, triglycerides and fibrinogen; for obesity adjusted for study, age, blood glucose, cholesterols, triglycerides and fibrinogen.

^4^ Mean fold change in parasitemia; change in parasitemia over 48 hr.

**Fig 2:**
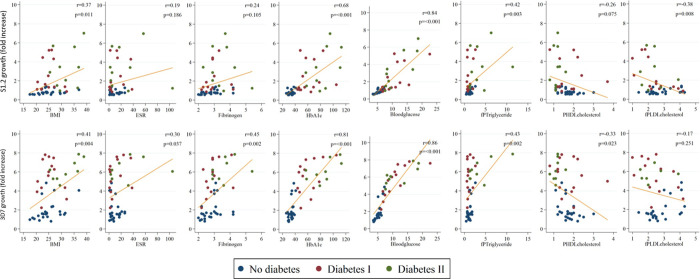


**Fig 3:**
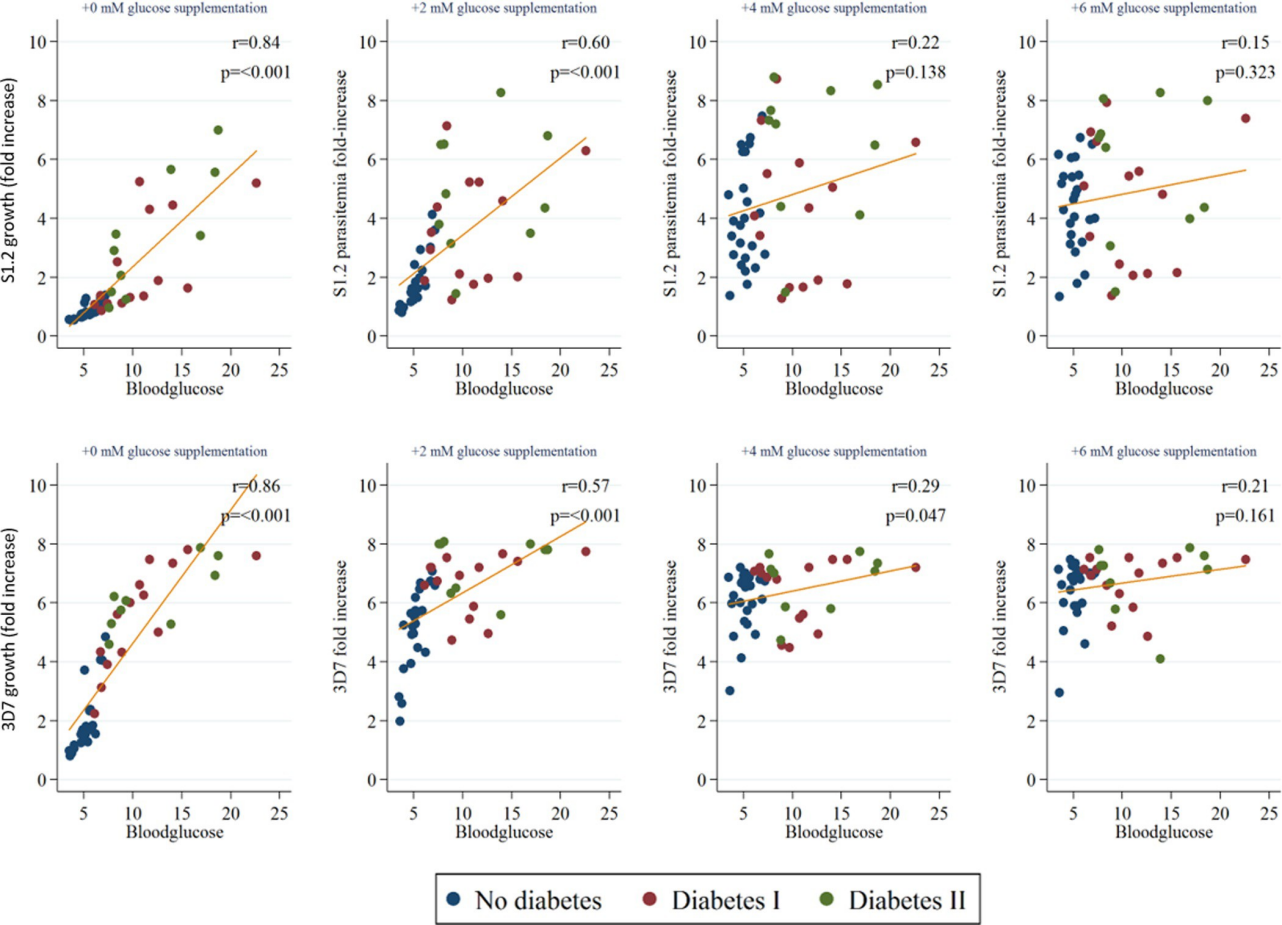


The continuous variables showing positive correlation with parasite growth (independent of rosetting) in both S1.2 and 3D7 assays include blood glucose (*P*<0.001 for both), HbA1c (*P*<0.001 for both), BMI (*P* = 0.010 and *P* = 0.002 respectively), fibrinogen (*P =* 0.007 and *P*<0.001 respectively) and triglycerides (*P* = 0.012 and *P* = 0.017 respectively) (S4 Table and Figs 2 and 3). Although obesity (BMI≥30 kg/m^2^) was not significantly associated with parasite growth in multivariable analyses (Table 3), BMI correlated with parasite growth in both S1.2 and 3D7 (*P* = 0.010 and *P* = 0.002 respectively) (S4 Table and Fig 2), probably due to loss of statistical power when using obesity (a categorical variable) as opposed to BMI (a continuous variable).

After adjusting for study, multivariable analyses showed that both type 1 and type 2 diabetes were still positively correlated to parasite growth in S1.2 (*P* = 0.003 and *P*<0.001 respectively, Fig 1B and Table 3) and 3D7 parasites (*P*<0.001 for both, Table 3). The association was still observed when additional adjustment for BMI and age was made (*P* = 0.023 and *P* = 0.002 for S1.2 grown in type 1 and type 2 respectively, and *P*<0.001 for both type 1 and type 2 in 3D7). However, after also adjusting for blood glucose, cholesterols, triglycerides and fibrinogen, only type 1 diabetes remained positively correlated to 3D7 parasite growth (*P* = 0.042 Table 3).

To exclude the effect of limiting glucose amounts, the same assay was also run with serum samples spiked with between 2 to 6 mM of glucose. In both S1.2 and 3D7 assays, there was a glucose concentration-dependent reduction in correlation between parasite growth and blood glucose, BMI, and triglycerides (Fig 3 and S4 Table). However, in 3D7 the correlation for fibrinogen and HbA1c with parasite growth was still significant after 6 mM glucose supplementation (*P* = 0.011 and *P* = 0.022 respectively, S4 Table). Since most correlations became insignificant after the supplementation of 6 mM glucose, we conclude that glucose concentration is the dominant factor influencing parasite growth.

## Discussion

The prevalence of diabetes and other metabolic diseases in the developing world has been increasing [1]. This development deserved particular attention in Sub-Saharan Africa where the double burdens of communicable and non-communicable diseases exert synergistically detrimental effects on human health [2, 37]. Considering this changing disease paradigm, the study of comorbidities is becoming increasingly relevant to the global healthcare landscape.

In this study, we show that erythrocytes from diabetic patients are more prone to forming rosettes when cultured with *P*. *falciparum*, and that elevated blood glucose levels allow parasites to establish higher parasitemia ex vivo.

These findings complement a previous retrospective study in Sweden involving 937 adults with imported malaria, where the association between comorbidities and severe *P*. *falciparum* malaria was investigated [8]. We observed that a higher proportion of patients with severe malaria had chronic diseases (30.4%), compared to the proportion with uncomplicated malaria (17.9%). Furthermore, the only non-communicable disease significantly associated with severe malaria in multivariable analyses was diabetes. Most diabetics in that study had type 2 diabetes so conclusions concerning type 1 could not be drawn. Among other host factors investigated, obesity was highly associated to severe malaria with over five times increased risk, independent of diabetes. The more common symptoms of severe malaria among the diabetic patients included respiratory distress with pulmonary edema, macroscopic hematuria and renal dysfunction. A non-significantly higher proportion of diabetics had hyperparasitemia (>5%) compared to non-diabetics. While the association of malaria severity with diabetes and obesity was clearly demonstrated, the biological basis for the association was not explored. The intent of the present study was to determine whether blood components from diabetics give rise to different *P*. *falciparum* rosetting and growth rates *ex vivo*.

Higher levels of parasite rosetting are associated with increasing severity of malaria [23, 24] and our findings show that parasites cultured in RBCs from diabetics had higher rosetting rates than those from the non-diabetic controls. This raises the possibility that the prevalence of severe malaria among type 2 diabetics observed previously [8] could be a consequence of augmented rosette formation. Interestingly, HbA1c, blood glucose and ESR correlated to rosetting rate even in the RBC-only assays where RBCs were washed free of serum components. This indicates that changes to the erythrocytes, and not differences in serum constitution, could account for augmented rosetting.

Several changes to the erythrocytes of diabetics have been well characterized. Firstly, prolonged hyperglycemia results in the glycation of RBC hemoglobin resulting in high HbA1c values, a clinical hallmark for poor glycemic control. Glycation also occurs on the erythrocyte membrane resulting in the formation of advanced glycation end products (AGEs) which increases binding to endothelial cells bearing receptors for AGEs [38]. Thirdly, there is also a loss of negatively charged sialic acid residues on RBC surface glycoproteins which reduces cell-cell repulsive forces and promotes cell aggregation [9]. It is conceivable that such aggregation-promoting factors could enhance rosette formation by complementing parasite adhesins such as PfEMP1s, RIFINs and STEVORS which are the primary mediators of rosette formation [27].

While elevated levels of acute phase proteins like fibrinogen and C-reactive protein in plasma play a critical role in rouleaux formation [17], we did not observe any association between C-reactive protein and rosetting rate in the serum-only assays. Although fibrinogen is an established mediator of rosetting [34] and the primary determinant of ESR [20], its effect here could not be tested since the serum-based assays are depleted of fibrinogen (converted to fibrin during blood clotting). The use of plasma for these assays was considered but many of these compounds that prevent coagulation (heparin, citrate and EDTA) are also known to interfere with parasite rosetting and growth [34] and would confound results.

Differences in the conclusions drawn from the RBC assays, serum assays and RBC+serum assays point towards the complex (multifactorial) nature of rosetting dependent on the interplay between pro-rosetting (such as the aforementioned RBC changes) and anti-rosetting factors (potentially including diabetes-related medications in serum, highlighted in the Results subsection on Parasite Rosetting). Determination of which factors dominate rosetting outcomes in vivo would very challenging.

In addition to increased parasite rosetting, serum from both type 1 and type 2 diabetes also gave rise to a higher level of parasite growth. Compared to the serum of healthy participants, serum from diabetic patients gave between 2.5- to 4-fold higher parasitemia after just one cycle of parasite replication. Blood glucose, HbA1c, BMI, fibrinogen and triglycerides all showed strong correlation with S1.2 and 3D7 parasite growth. In order to find out if HbA1c, BMI, fibrinogen or triglycerides can affect parasite growth independent of glucose levels, we supplemented serum samples with exogenous glucose at the start of the assay. The near complete loss of correlations indicate that glucose levels are the main determinant of parasite growth ex vivo, even in serum that is nutrient replete. This result is in agreement with a previous study showing that in vitro parasite growth is highly dependent on glucose levels [35]. As such, a possible basis for the association between diabetes and severe malaria is that higher blood glucose levels promote parasite growth, leading to hyperparasitemia and severe malaria. However, our previous study showed that diabetes was associated to severe manifestations independent of hyperparasitemia [8] and thus increased rosette formation may offer a more robust explanation.

There was a total of ten obese individuals in this study but too few non-diabetic obese to be able to draw any definitive conclusions concerning the role of obesity in the pathogenesis of severe malaria. Although we could not show any positive association between obesity and rosetting rate, there was a positive correlation between BMI and parasitemia also after addition of glucose (in the S1.2 assay up to 4mM, S4 Table). Interestingly, a significantly higher proportion of obese patients in the retrospective comorbidity study had hyperparasitemia (both >5% and >10%) [8]. Taken together, results from epidemiological as well as the present experimental study suggest that serum factors affecting parasite growth could partly explain the association between obesity and severe malaria.

In our 48 hr microtiter plate-based assays for parasite growth, hematocrit was kept at 4% and start parasitemia at 1.5%. It might be argued that this static experimental design is the reason why glucose emerges as a factor limiting parasite growth and that glucose will not be the limiting factor in the well-equilibrated physiological environment. One review has estimated that each microliter of normal plasma contains between 60 to 600 times more glucose than what each *P*. *falciparum* parasite requires [39]. However, this does not take into account the number of parasites supported by each microliter of plasma–approximately 50,000 parasites per microliter of blood (assuming 1% parasitemia and 5 million RBCs per microliter of blood). Especially in a low-perfusion microenvironment characteristic of complicated malaria, it is very likely that glucose levels are limiting. The clinical hallmark of hypoglycemia in severe malaria, especially in children, is further evidence of the importance of glucose in disease severity. Taken together, elevated blood glucose could potentially enhance parasite growth in diabetic patients.

Based on the glucose supplementation experiments, the effect of glucose on parasite growth was demonstrated to be causative. However, the other associations highlighted in this study are only correlative and may not be the biological basis for the observed differences in rosetting rate or parasite growth. Controlled experiments will need to be performed to distinguish between associated and causal factors, with subsequent validation on multiple laboratory and clinical parasite strains performed to strengthen the relevance of these findings. The level of AGEs and sialic acid present on RBC surface, as well as the effect of fibrinogen, represent potentially important factors influencing parasite rosetting that have not been investigated in this study. This is especially since previous studies have shown that serum proteins like albumin, fibrinogen, IgM and von Willebrand factor are involved in rosetting [34]. Additionally, the serendipitous finding that serum from type 2 diabetics correlate negatively with rosetting suggests that serum factors or medications from this group could reduce rosetting.

In conclusion, this ex vivo study suggests a potential biological basis for the comorbidity of diabetes and severe malaria; with blood parameters characteristic of uninfected type 1 and type 2 diabetics (namely blood glucose, HbA1c and ESR) shown to correlate with increased parasite rosetting and growth rates. In particular, the findings that increased blood glucose concentrations augment parasite growth suggests that careful regulation of blood sugar could complement conventional antimalarial therapy in reducing parasitemia. A such, the present WHO guideline regarding the monitoring of blood glucose to prevent hypoglycemia, could be modified to restrict hyperglycemia as well. Additionally, since changes to erythrocytes are attributed to increased rosetting, the practice of good long-term glycemic control could prove doubly beneficial in reducing both diabetic and malaria morbidity and mortality. As such, the implementation of policies to diagnose diabetes and promote good glycemic control should be emphasized in malaria-endemic regions especially.

### Limitations of the study

This pilot study involves a small sample of 25 diabetics and 25 non-diabetic controls, which limits the strength of conclusions. Stratification to further investigate certain specific questions was therefore not always possible. Fibrinogen, an established mediator of rosetting and determinant of ESR could not be assayed due to experimental design constraints (coagulation). The relevance of ex vivo assays also inherently questions about physiological relevance, but the nature of malaria infection, and in this case the association with severe malaria pathology, inherently restricts this study of a human-specific malaria to ex vivo/in vitro models. This study only makes use of a single rosetting parasite (FCR3S1.2) which may not be representative of other rosetting strains/isolates with highly heterogenic rosetting antigens. Other limitations have been elaborated in the Discussion.

## Resource availability

### Lead contact

Further information and requests for resources and reagents should be directed to and will be fulfilled by the Lead Contact, Mats Wahlgren (mats.wahlgren@ki.se).

### Materials availability

There are restrictions to the availability of patient erythrocytes and serum due to the minimal amount of material that was drawn. This study did not generate other new unique reagents.

### Data and code availability

The published article includes all datasets generated or analyzed during this study. Original/source data for flow cytometry files (fcs files and gating parameters) used in the paper is available from the Lead upon request.

## Methods

### Study population and clinical parameters

Adult subjects with diabetes (type 1 and type 2) undergoing a diabetes management course at Karolinska University Hospital, as well as non-diabetic control volunteers from Stockholm, Sweden, were invited to participate in the study. Recruitment and sampling were performed during scheduled routine visits at the Department of Endocrinology, from different donors at in June 2016 (Study 1) and in May 2017 (Study 2). Individuals with fever or bloodborne infection (chronic hepatitis or HIV) were excluded.

Details of the participants’ medical history including previous malaria, travel history, ongoing medication, weight and height were collected by research nurses. Intravenous blood was drawn for routine blood analyses at the Department of Clinical Chemistry at Karolinska University Hospital including hematocrit, MCV of the erythrocyte, glucose, HbA1C, C-reactive protein (CRP), erythrocyte sedimentation rate (ESR), fibrinogen, apolipoprotein B and blood lipids (cholesterol, LDL, HDL, triglyceride) as well as for ABO blood group typing.

Body mass index (BMI) was calculated as weight (kilograms) divided by square height (meters), and categorised according to WHO’s BMI classification for adults [40]. Glycemic control was defined as poor if HbA1c ≥53 mmol/mol. Diabetes-related complications were ranked as mild, moderate or severe depending on the number and type complications described in the patient questionnaire (S1 Data).

### Sampling for experiments

Intravenous blood for *ex vivo* experiments were collected in BD Vacutainer® Citrate tubes (for RBC collection) and BD Vacutainer® SST™ tubes (for serum collection). For RBC collection, citrate tubes were spun down, plasma and buffy coat removed, and RBCs washed twice with RPMI 1640 before being stored at 4°C. For serum collection, STT tubes were spun down, serum decanted into a new tube and heat inactivated at 56°C for 1 hr before being stored at -80°C. Serum was used in all assays because heparin and citrate (used to prevent blood coagulation for the isolation of plasma) affects parasite reinvasion and rosetting [22, 34, 41]. All samples were processed and stored on the day of collection and RBCs were assayed within a week of collection.

### Parasite cultures

Laboratory-adapted *P*. *falciparum* cultures of rosetting FCR3S1.2 (S1.2, rosetting rate maintained >75%) and non-rosetting 3D7 were grown by standard methods at 2% hematocrit (O+ erythrocytes) in RMPI1640 supplemented with 10% A+ human serum, at 37°C, with shaking, and under mixed-gas conditions (5% O_2_, 5% CO_2_ and 90% N_2_) [42]. The blood group O+ erythrocytes and A+ serum that was used for routine parasite culture, while AB+ serum was used for rosetting rate assays of clinical samples to prevent agglutination of RBCs from patients with B and AB blood groups. These were obtained from the Karolinska University Hospital Blood Bank, collected as part of routine donations and completely de-identified before accessed. Cultures routinely tested negative for mycoplasma contamination. Sorbitol synchronization was carried out weekly and Ficoll-enrichment was performed as necessary to select for rosetting S1.2 parasites [43].

### Rosetting rate assays

Late-stages of S1.2 parasites were enriched by mechanical disruption of rosettes (needle trituration) followed by magnetic activated cell sorting [43]. The resulting pellet of enriched trophozoites/schizonts (parasitemia in excess of 75%) was used immediately for rosetting assays. Non-rosetting 3D7 was not included in these assays.

In assays using RBCs from the participants (RBC assay), each microtiter well contained 1 μl of participants’ RBCs and 0.01 μl of MACS-enriched parasites, together with 90 μl of RPMI supplemented with 10 μl of AB+ serum. The proportion of serum used in this assay (10%) is identical to routine cultures.

In assays using serum from participants (serum assay), each well contained 50 μl of participants’ serum together with 50 μl RPMI, 1 μl O+ RBCs and 0.01 μl MACS-enriched parasites. The proportion of serum used in this assay (50%) is higher than routine culture media so as to minimize dilution of serum factors which may affect rosetting. 50% RPMI was still necessary as excessively high proportions of serum results in restricted parasite growth (see Results).

In assays using both RBC and serum from participants (RBC+serum assay), each well contained 1 μl of donor RBC, 50 μl of serum from the same donor, 50 μl RPMI and 0.01 μl MACS-enriched parasites. This combination was used to assess if the combination of autologous RBC and serum would result in a different effect on parasite rosetting than these components separately.

After setting up the assays, microtiter plates were left for 48 hrs in a dark humidified chamber perfused with mixed gas (without shaking) at 37°C prior to parasite staining and flow cytometry.

### Parasite growth assays and spiking of serum with exogenous glucose

The parasitemia of ring stage cultures of S1.2 and 3D7 were quantified by cytometry on the day of experiment and adjusted to 1.5% starting parasitemia with uninfected erythrocytes. Cells were spun down and 2.5 μl of packed cells were added to wells containing 45 μl of donor serum and 5 μl of PBS that contained 0, 20, 40 or 60 mM of glucose. The proportion of serum in these assays is approximately 85% (no RPMI was added) so as to best observe the effects of serum components on parasite growth. Plates were incubated for 48 hrs in a dark humidified chamber perfused with mixed gas (without shaking) at 37°C prior to parasite staining and flow cytometry.

### Cell staining and flow cytometry

A 10× solution of Hoechst (100 μg/ml) and dihydroethidium (60 μg/ml) was used to stain cells, and BD FACSVerse with plate reader was used to measure parasitemia and percentage multiplets as detailed elsewhere [42]. The percentage multiplets has been shown to correlate with rosetting rate and is used here as a proxy to indicate the relative rosetting rate [42, 44, 45]. The proportion of cells being gated and used in analyses are all presented in S2 Data.

### Statistical analyses

Unless otherwise indicated, all data shown is presented as means with standard deviation (SD). All analyses were done with Stata version 13. The outcomes were S1.2 rosetting rate (as measured in RBC, serum and RBC+serum assays) and S1.2 and 3D7 parasitemia. We used linear regression with robust standard errors [46]. Rosetting rate assays were carried out separately for Study 1 and 2 owing to the requirement for fresh RBCs for the assays and the interval between studies. As a result, analyses were done with both datasets separated or merged to take into account potential inter-experimental variability. Multivariable analyses were performed on merged datasets adjusted for study, or adjusted for study, age and BMI, and finally with additional adjustment for glucose and ESR. In parasite growth assays, stored (frozen) serum from both study 1 and 2 were run in the same experiment and analyzed as a merged dataset. Multivariable analyses were performed adjusted for same factors as above, but with final additional adjustment for glucose, cholesterols and triglycerides (factors which may affect parasite growth in vitro [35, 47]), as well as fibrinogen. Univariate associations between S1.2 and 3D7 parasitemia (with added glucose and explanatory variables) were investigated using Spearman’s ρ. As a sensitivity analysis, we display these associations graphically using standard linear regression techniques (trend lines and Pearson’s r) in Figs 2 and 3.

### Ethical considerations

All experiments were performed with written informed consent in accordance with relevant ethical guidelines and regulations. This study was approved by the Regional Ethical Review Board in Stockholm, Sweden (2006/893-31/4, 2015/2200-32, 2016/1940-32 and 2009/668-31/3).

## Supporting information

S1 Table Characteristics of the study population. (DOCX)

S2 Table Association of variables with rosetting rate in red blood cell (RBC), serum and RBC+serum assay. (DOCX)

S3 Table Association of diabetes and obesity with rosetting rate in multivariable analyses (Study 1+2). (DOCX)

S4 Table Correlation of variables and parasite growth after glucose supplementation (Study 1+2). (DOCX)

S1 Data (XLSX)

S2 Data (XLSX)
