# Supplementary material for: Enhanced virulence of Plasmodium falciparum in blood of diabetic patients
Source: PLoS One. 2021 Jun 17;16(6):e0249666. doi: 10.1371/journal.pone.0249666 (PMC8211161; doi:10.1371/journal.pone.0249666)
Supplement: S1 Table — (DOCX) [file pone.0249666.s001.docx]

**S1 Table. Characteristics of the study population**

| **Group**  Subgroup | **Study1+2** | | | | **Study 1** | | | **Study 2** | | | |
| --- | --- | --- | --- | --- | --- | --- | --- | --- | --- | --- | --- |
|  | **All** | **Healthy** | **Type 1** | **Type 2** | **All** | **Healthy** | **Type 1** | **All** | **Healthy** | **Type 1** | **Type 2** |
| **Participants, n (%)** | 50 (100) | 25 (50) | 15 (30) | 10 (20) | 21 (100) | 9 (43) | 12 (57) | 29 (100) | 16 (55) | 3 (10) | 10 (34) |
| **Sex (female), n (%)** | 31 (62) | 19 (76) | 8 (53) | 4 (40) | 15 (71) | 8 (89) | 7 (58) | 16 (55) | 11 (69) | 1 (33) | 4 (40) |
| **Age (yrs), mean [SD)** | 43.2 [13.5] | 40.4 [12.5] | 38.4 [12.1] | 57.3 [8.6] | 42.5 [12.6] | 49.1 [13.3] | 37.6 [9.8] | 43.7 [14.4] | 35.5 [9.2] | 41.7 [21.6] | 57.3 [8.6] |
| **BMI (kg/m^2^), mean [SD]** | 26.32 [5.0] | 24.76 [4.4] | 25.26 [3.6] | 31.81 [4.7] | 25.18 [3.4] | 25.89 [3.5] | 24.65 [3.4] | 27.14 [5.8] | 24.13 [4.8] | 27.68 [3.9] | 31.81 [4.7] |
| **BMI classification^1^, n (%)** |  |  |  |  |  |  |  |  |  |  |  |
| Underweight | 1 (2) | 1 (4) | 0 (0) | 0 (0) | 0 (0) | 0 (0) | 0 (0) | 1 (3) | 1 (6) | 0 (0) | 0 (0) |
| Normal | 23 (26) | 15 (60) | 8 (53) | 0 (0) | 12 (57) | 5 (55) | 7 (58) | 11 (38) | 10 (62) | 1 (33) | 0 (0) |
| Overweight | 16 (32) | 6 (24) | 5 (33) | 5 (50) | 6 (29) | 2 (22) | 4 (33) | 10 (34) | 4 (25) | 1 (33) | 5 (50) |
| Obese | 10 (20) | 3 (12) | 2 (13) | 5 (50) | 3 (14) | 2 (22) | 1 (8) | 7 (24) | 1 (6) | 1 (33) | 5 (50) |
| **Blood group, n (%)** |  |  |  |  |  |  |  |  |  |  |  |
| O | 20 (40) | 10 (40) | 7 (47) | 3 (30) | 8 (38) | 2 (22) | 6 (50) | 12 (41) | 8 (50) | 1 (33) | 3 (30) |
| A | 21 (42) | 11 (44) | 6 (40) | 4 (40) | 9 (43) | 5 (56) | 4 (33) | 12 (41) | 6 (38) | 2 (67) | 4 (40) |
| B | 5 (10) | 2 (8) | 2 (13) | 1 (10) | 2 (10) | 0 (0) | 2 (17) | 3 (10) | 2 (13) | 0 (0) | 1 (10) |
| AB | 4 (8) | 2 (8) | 0 (0) | 2 (20) | 2 (10) | 2 (22) | 0 (0) | 2 (7) | 0 (0) | 0 (0) | 2 (20) |
| **Clinical chemistry, mean [SD]** |  |  |  |  |  |  |  |  |  |  |  |
| HbA1c (mmol/mol) | 52.5 [24.1] | 33.6 [3.7] | 63.1 [17.5] | 83.8 [19.4] | 52.4 [20.7] | 35.4 [4.1] | 65.2 [18.8] | 52.5 [26.7] | 32.5 [3.1] | 55.0 [8.9] | 83.8 [19.4] |
| Blood glucose (mM) | 8.3 [4.5] | 5.2 [1.0] | 11.1 [4.4] | 11.8 [4.7] | 9.1 [4.7] | 5.6 [0.9] | 11.7 [4.6] | 7.7 [4.3] | 4.9 [1.0] | 8.6 [2.0] | 11.8 [4.7] |
| Hematocrit (x10^12^/L) | 4.3 [0.6] | 4.2 [0.3] | 4.7 [0.8] | 4.2 [0.5] | 4.6 [0.7] | 4.3 [0.4] | 4.9 [0.8] | 4.1 [0.4] | 4.1 [0.3] | 4.2 [0.6] | 4.2 [0.5] |
| MCV (fL) | 87.6 [5.6] | 89.5 [4.1] | 86.0 [6.7] | 85.2 [5.7] | 88.4 [6.1] | 91.1 [2.1] | 86.3 [7.4] | 87.0 [5.1] | 88.6 [4.8] | 84.7 [3.2] | 85.2 [5.7] |
| ESR (mm/hr) | 14.5 [18.5] | 9.8 [7.8] | 14.1 [15.8] | 26.6 [33.0] | 14.2 [14.2] | 12.4 [9.8] | 15.5 [17.1] | 14.7 [21.3] | 8.4 [6.2] | 8.7 [9.1] | 26.6 [33.0] |
| Fibrinogen (g/L)^2^ | 3.0 [0.7] | 2.8 [0.6] | 3.00 [0.6] | 3.5 [0.9] | 3.1 [0.7] | 3.1 [0.7] | 3.0 [0.7] | 3.0 [0.7] | 2.7 [0.4] | 2.8 [0.4] | 3.5 [0.9] |
| C-react. protein (mg/L) | 3.2 [4.4] | 2.5 [4.13] | 3.7 [5.5] | 4.0 [2.9] | 4.1 [5.8] | 3.9 [5.8] | 4.3 [6.1] | 2.5 [2.9] | 1.8 [2.7] | 1.3 [0.6] | 4.0 [2.9] |
| Apolipoprotein B (g/L)^2^ | 0.9 [0.3] | 0.9 [0.2] | 0.8 [0.3] | 1.1 [0.4] | 0.7 [0.3] | N.D. | 0.7 [0.3] | 0.9 [0.3] | 0.9 [0.2] | 0.8 [0.4] | 1.1 [0.4] |
| Triglycerides (mM) | 1.7 [1.8] | 1.2 [0.5] | 1.2 [0.6] | 3.8 [3.2] | 1.3 [0.6] | 1.5 [0.5] | 1.1 [0.5] | 2.0 [2.3] | 1.0 [0.4] | 1.4 [1.1] | 3.8 [3.2] |
| Cholesterol (mM) | 5.0 [1.0] | 5.2 [0.9] | 4.7 [1.0] | 5.1 [1.4] | 5.1 [1.1] | 5.6 [1.1] | 4.7 [1.0] | 4.9 [1.0] | 4.9 [0.7] | 4.5 [1.3] | 5.1 [1.4] |
| HDL-cholesterol (mM) | 1.6 [0.6] | 1.8 [0.4] | 1.7 [0.7] | 1.2 [0.2] | 1.8 [0.6] | 1.7 [0.3] | 1.8 [0.7] | 1.5 [0.5] | 1.8 [0.5] | 1.5 [0.8] | 1.2 [0.2] |
| LDL cholesterol (mM)^2^ | 2.6 [0.9] | 2.8 [0.8] | 2.4 [0.9] | 2.3 [0.8] | 2.8 [0.9] | 3.2 [0.9] | 2.4 [0.8] | 2.5 [0.8] | 2.6 [0.7] | 2.4 [1.6] | 2.3 [0.8] |
| LDL/HDL | 1.8 [0.8] | 1.7 [0.7] | 1.6 [0.8] | 2.2 [1.1] | 1.7 [0.7] | 1.9 [0.6] | 1.5 [0.7] | 1.8 [0.9] | 1.6 [0.7] | 2.0 [1.3] | 2.2 [1.1] |

^1^ WHO BMI classification for adults BMI <18.5 (underweight), 18.5-25 (normal), 25-30 (overweight), >30 obese. ^2^ Missing patient parameters (cells with missing values have been shaded green in Supplementary Data S1): apolipoprotein B was not measured for all nine healthy controls in Study 1 and from one control in Study 2, fibrinogen levels for one type 1 diabetic in Study 2 was missing and LDL-cholesterol could not be measured in two type 2 diabetics in Study 2 due to high glycerol levels that interfered with measurements.
