# Supplementary material for: Enhanced virulence of Plasmodium falciparum in blood of diabetic patients
Source: PLoS One. 2021 Jun 17;16(6):e0249666. doi: 10.1371/journal.pone.0249666 (PMC8211161; doi:10.1371/journal.pone.0249666)
Supplement: S2 Table — (DOCX) [file pone.0249666.s002.docx]

**S2 Table. Association of variables with rosetting rate in red blood cell (RBC), serum and RBC+serum assay**

| **Assay** | **Variable** | **Study 1** | | | | **Study 2** | | | |
| --- | --- | --- | --- | --- | --- | --- | --- | --- | --- |
|  |  | **Rosetting**  **mean % multiplets [SD]** | **Coef.** | **95% CI** | ***P*** | **Rosetting**  **mean % multiplets [SD]** | **Coef.** | **95% CI** | ***P*** |
| **RBC** | Non-diabetic | 18.03 [2.74] | 0 |  |  | 17.11 [5.76] | 0 |  |  |
|  | Diabetes type 1 | 20.98 [4.02] | 2.95 | -0.29;6.19 | 0.072 | 22.63 [3.19] | 5.52 | -0.04;11.09 | 0.052^2^ |
|  | Diabetes type 2 |  |  |  |  | 25.59 [6.76]^1^ | 8.48 | 2.68;14.28 | 0.006^3^ |
|  | Non-obese | 20.20 [3.85] | 0 |  |  | 20.05 [7.22] | 0 |  |  |
|  | Obese | 16.84 [0.61] | -3.36 | -5.51;-1.21 | 0.004 | 21.8 [6.43] | 1.75 | -4.99;8.49 | 0.598 |
|  | Male | 18.58 [2.02] | 0 |  |  | 20.34 [7.84] | 0 |  |  |
|  | Female | 20.18 [4.23] | 1.60 | -1.43;4.63 | 0.284 | 20.49 [6.53] | 0.16 | -5.81;6.12 | 0.957 |
|  | Age |  | -0.07 | -0.20;0.05 | 0.212 |  | 0.18 | 0.01;0.35 | 0.044 |
|  | Blood group O | 22.90 [1.97] | 0 |  |  | 17.47 [6.10] | 0 |  |  |
|  | Blood group A | 16.84 [1.06] | -6.06 | -7.82;-4.30 | <0.001 | 22.70 [7.67] | 5.23 | -1.05;11.51 | 0.099 |
|  | Blood group B | 22.83 [7.37] | -0.08 | -15.7;15.54 | 0.992 | 21.11 [6.95] | 3.64 | -7.19;14.48 | 0.494 |
|  | Blood group AB | 16.83 [0.33] | -6.07 | -7.79;-4.35 | <0.001 | 24.67 [4.67] | 7.20 | -3.15;17.55 | 0.164 |
|  | Blood glucose (mM) |  | 4.59 | 1.31;7.88 | 0.009 |  | 7.07 | 1.94;12.20 | 0.009 |
|  | ESR (mm/Hr) |  | 1.24 | -0.03;2.50 | 0.056 |  | 2.31 | 0.34;4.28 | 0.023 |
|  | HbA1c (mmol/mol) |  | 3.65 | -1.50;8.80 | 0.154 |  | 9.16 | 3.65;14.67 | 0.002 |
|  | Fibrinogen (g/L) |  | 1.80 | -0.73;4.33 | 0.153 |  | 3.07 | 0.55;5.59 | 0.019 |
|  | Triglycerides (mM) |  | -0.35 | -5.67;4.98 | 0.893 |  | 3.25 | -0.80;7.29 | 0.111 |
|  | Cholesterol (mM) |  | -5.70 | -14.61;3.21 | 0.197 |  | 3.22 | -10.51;16.95 | 0.634 |
| **Serum** | Non-diabetic | 19.34 [0.97] |  |  |  | 33.99 [2.01] |  |  |  |
|  | Diabetes type 1 | 18.96 [0.71] | -0.38 | -1.24;0.48 | 0.368 | 35.38 [0.93] | 1.39 | -0.36;3.14 | 0.114^2^ |
|  | Diabetes type 2 |  |  |  |  | 31.03 [2.99] | -2.95 | -5.29;-0.62 | 0.015^3^ |
|  | Non-obese | 19.02 [0.69] |  |  |  | 33.11 [2.18] |  |  |  |
|  | Obese | 19.73 [1.48] | 0.70 | -1.52;2.92 | 0.514 | 33.00 [4.36] | -0.10 | -3.89;3.69 | 0.956 |
|  | Male | 19.00 [0.79] | 0 |  |  | 33.84 [2.26] | 0 |  |  |
|  | Female | 19.19 [0.88] | 0.18 | -0.71;1.08 | 0.673 | 32.42 [3.09] | -1.41 | -3.58;0.75 | 0.191 |
|  | Age |  | 0.03 | 0.002;0.07 | 0.037 |  | -0.05 | -0.15;0.05 | 0.301 |
|  | Blood group O | 19.00 [0.79] | 0 |  |  | 33.38 [2.89] | 0 |  |  |
|  | Blood group A | 19.26 [0.95] | 0.26 | -0.73;1.25 | 0.587 | 33.32 [2.08] | -0.06 | -2.31;2.19 | 0.957 |
|  | Blood group B | 18.69 [0.18] | -0.31 | -1.09;0.48 | 0.419 | 34.52 [0.61] | 1.14 | -0.87;3.15 | 0.252 |
|  | Blood group AB | 19.47 [1.13] | 0.47 | -2.01;2.96 | 0.691 | 27.8 [3.11] | -5.58 | -12.25;1.09 | 0.097 |
|  | Blood glucose (mM) |  | -0.39 | 1.32;0.54 | 0.393 |  | -1.92 | -4.58;0.73 | 0.148 |
|  | ESR (mm/Hr) |  | 0.10 | -0.26;0.46 | 0.577 |  | -0.48 | -1.82;0.87 | 0.474 |
|  | HbA1c (mmol/mol) |  | -0.51 | -1.63;0.62 | 0.357 |  | -2.74 | -5.48;0.00 | 0.050 |
|  | Fibrinogen (g/L) |  | 0.46 | -0.49;1.42 | 0.322 |  | -0.28 | -2.81;2.25 | 0.819 |
|  | Triglycerides (mM) |  | 0.28 | -0.58;1.15 | 0.499 |  | -2.34 | -3.72;-0.95 | 0.002 |
|  | Cholesterol (mM) |  | 2.69 | 1.24;4.14 | 0.001 |  | -4.89 | -11.46;1.68 | 0.138 |
| **RBC+serum** | Non-diabetic | 19.90 [1.36] |  |  |  | 23.74 [4.15] |  |  |  |
|  | Diabetes type 1 | 22.52 [3.51] | 2.62 | 0.07;5.16 | 0.044 | 25.08 [11.81] | 1.34 | -16.04; 18.72 | 0.875^2^ |
|  | Diabetes type2 |  |  |  |  | 25.44 [7.97] | 1.71 | -4.54;7.96 | 0.578^3^ |
|  | Non-obese | 21.50 [3.16] |  |  |  | 24.13 [5.68] |  |  |  |
|  | Obese | 20.42 [2.16] | -1.08 | -4.69;2.53 | 0.538 | 25.59 [8.82] | 1.47 | -7.07; 10.00 | 0.727 |
|  | Male | 20.47 [2.21] | 0 |  |  | 23.02 [8.39] | 0 |  |  |
|  | Female | 21.71 [3.30] | 1.24 | -1.59;4.07 | 0.369 | 25.60 [4.02] | 2.58 | -3.08;8.24 | 0.356 |
|  | Age |  | -0.07 | -0.15;0.001 | 0.052 |  | 0.16 | -0.03;0.35 | 0.100 |
|  | Blood group O | 22.03 [1.74] | 0 |  |  | 23.76 [6.16] | 0 |  |  |
|  | Blood group A | 20.26 [1.31] | -1.77 | -3.57;0.03 | 0.053 | 25.17 [5.82] | 1.41 | -4.15;6.96 | 0.606 |
|  | Blood group B | 25.83 [8.45] | 3.80 | -14.18;21.78 | 0.660 | 29.12 [9.01] | 5.36 | -8.37;19.09 | 0.427 |
|  | Blood group AB | 19.33 [2.57] | -2.70 | -8.34;2.96 | 0.327 | 18.05 [1.72] | -5.71 | -10.95;-0.47 | 0.034 |
|  | Blood glucose (mM) |  | 3.17 | -0.29;6.64 | 0.070 |  | -1.11 | -8.04;5.83 | 0.745 |
|  | ESR (mm/Hr) |  | 0.55 | -0.40;1.50 | 0.238 |  | 1.55 | -1.36;4.46 | 0.284 |
|  | HbA1c (mmol/mol) |  | 2.85 | 0.49;5.22 | 0.021 |  | 3.06 | -3.96;10.08 | 0.377 |
|  | Fibrinogen (g/L) |  | 0.17 | -1.49;1.83 | 0.836 |  | 0.99 | -7.35;9.32 | 0.809 |
|  | Triglycerides (mM) |  | -1.89 | -5.76;1.98 | 0.319 |  | -2.55 | -5.08;-0.01 | 0.049 |
|  | Cholesterol (mM) |  | -3.51 | -8.91;1.89 | 0.189 |  | 1.97 | -15.38;19.31 | 0.817 |

¹ RBCs from one type 2 diabetic (Donor 6.27 on 14 different of medications) showed atypical forward- and side-scatter profiles on the cytometer and accurate determination of the percentage of multiplets was not possible. ² Type I diabetics compared with non-diabetics. ^3^ Type II diabetics compared with non-diabetics.
