# Supplementary material for: Enhanced virulence of Plasmodium falciparum in blood of diabetic patients
Source: PLoS One. 2021 Jun 17;16(6):e0249666. doi: 10.1371/journal.pone.0249666 (PMC8211161; doi:10.1371/journal.pone.0249666)
Supplement: S3 Table — (DOCX) [file pone.0249666.s003.docx]

**S3 Table. Association of diabetes and obesity with rosetting rate in multivariable analyses (Study 1+2)**

| **Assay** | **Variable** | **Adjusted^1^** | | | **Adjusted^2^** | | | **Adjusted^3^** | | |
| --- | --- | --- | --- | --- | --- | --- | --- | --- | --- | --- |
|  |  | **Coef.** | **95% CI** | ***P*** | **Coef.** | **95% CI** | ***P*** | **Coef.** | **95% CI** | ***P*** |
| **RBC** | Non-diabetic | 0 |  |  | 0 |  |  | 0 |  |  |
|  | Diabetes type 1 | 3.80 | 1.28;6.32 | 0.004 | 3.76 | 0.21;7.31 | 0.038 | 3.60 | -0.93;7.59 | 0.076 |
|  | Diabetes type 2 | 8.21 | 3.00;13.41 | 0.003 | 8.69 | 2.07;15.32 | 0.011 | 8.39 | 1.37;15.40 | 0.020 |
|  | Non-obese | 0 |  |  | 0 |  |  | 0 |  |  |
|  | Obese | -0.05 | -4.18;4.07 | 0.979 | -1.03 | -5.68;3.61 | 0.656 | -2.11 | -5.97;1.75 | 0.276 |
| **Serum** | Non-diabetic | 0 |  |  | 0 |  |  | 0 |  |  |
|  | Diabetes type 1 | 0.22 | -0.61;1.04 | 0.601 | 0.46 | -.31;1.23 | 0.233 | 0.43 | -1.11;1.97 | 0.576 |
|  | Diabetes type 2 | -3.15 | -5.27;-1.03 | 0.005 | -4.59 | -7.03;-2.15 | <0.001 | -4.59 | -7.12;-2.06 | 0.001 |
|  | Non-obese | 0 |  |  |  |  |  | 0 |  |  |
|  | Obese | 0.16 | -2.13;2.45 | 0.888 | 0.40 | -2.25;3.05 | 0.760 | 0.80 | -1.79;3.39 | 0.536 |
| **RBC+serum** | Non-diabetic | 0 |  |  | 0 |  |  | 0 |  |  |
|  | Diabetes type 1 | 2.19 | -2.11;6.49 | 0.310 | 3.04 | -1.58-7.65 | 0.191 | 4.96 | -0.55;10.48 | 0.077 |
|  | Diabetes type2 | 1.85 | -3.93;7.63 | 0.522 | 0.59 | -5.82-7.00 | 0.853 | 2.53 | -4.27;9.33 | 0.456 |
|  | Non-obese | 0 |  |  | 0 |  |  | 0 |  |  |
|  | Obese | 0.57 | -4.30;5.43 | 0.815 | -.29 | -5.82;5.25 | 0.917 | -.49 | -6.50;5.51 | 0.869 |

^1^ Adjusted for study. ^2^ For diabetes adjusted for study, age and BMI; for obesity adjusted for study and age. ^3^ For diabetes adjusted for study, age, BMI, B-glucose and ESR; for obesity adjusted for study, age, B-glucose and ESR.
