# Supplementary material for: Enhanced virulence of Plasmodium falciparum in blood of diabetic patients
Source: PLoS One. 2021 Jun 17;16(6):e0249666. doi: 10.1371/journal.pone.0249666 (PMC8211161; doi:10.1371/journal.pone.0249666)
Supplement: S4 Table — (DOCX) [file pone.0249666.s004.docx]

**S4 Table.** **Correlation of variables and parasite growth after glucose supplementation (Study 1+2)**

| **Variable** | **Glucose spike**  **(mM)** | **S1.2 Growth** | | **3D7 Growth** | |
| --- | --- | --- | --- | --- | --- |
|  |  | **Spearman’s rho** | ***P*** | **Spearman’s rho** | ***P*** |
| **Blood glucose (mM)** | 0 | 0.913 | <0.001 | 0.950 | <0.001 |
|  | 2 | 0.740 | <0.001 | 0.690 | <0.001 |
|  | 4 | 0.208 | 0.157 | 0.305 | 0.037 |
|  | 6 | 0.111 | 0.454 | 0.264 | 0.073 |
| **HbA1c (mmol/mol)** | 0 | 0.779 | <0.001 | 0.841 | <0.001 |
|  | 2 | 0.650 | <0.001 | 0.718 | <0.001 |
|  | 4 | 0.307 | 0.037 | 0.456 | 0.001 |
|  | 6 | 0.215 | 0.142 | 0.307 | 0.022 |
| **BMI (kg/m^2^)** | 0 | 0.368 | 0.010 | 0.437 | 0.002 |
|  | 2 | 0.455 | 0.001 | 0.523 | <0.001 |
|  | 4 | 0.309 | 0.017 | 0.238 | 0.107 |
|  | 6 | 0.147 | 0.317 | 0.233 | 0.114 |
| **ESR (mm/hr)** | 0 | 0.072 | 0.629 | 0.189 | 0.203 |
|  | 2 | 0.018 | 0.903 | 0.210 | 0.156 |
|  | 4 | 0.031 | 0.833 | 0.333 | 0.022 |
|  | 6 | 0.029 | 0.846 | 0.149 | 0.318 |
| **Fibrinogen (g/L)** | 0 | 0.387 | 0.007 | 0.500 | <0.001 |
|  | 2 | 0.327 | 0.025 | 0.466 | 0.001 |
|  | 4 | 0.121 | 0.419 | 0.347 | 0.018 |
|  | 6 | -0.023 | 0.877 | 0.371 | 0.011 |
| **Triglycerides (mM)** | 0 | 0.361 | 0.012 | 0.346 | 0.017 |
|  | 2 | 0.020 | 0.336 | 0.266 | 0.070 |
|  | 4 | 0.066 | 0.267 | 0.140 | 0.347 |
|  | 6 | 0.143 | 0.215 | 0.250 | 0.091 |
| **Cholesterol (mM)** | 0 | -0.124 | 0.400 | -0.079 | 0.598 |
|  | 2 | -0.186 | 0.205 | -0.064 | 0.700 |
|  | 4 | -0.088 | 0.551 | -0.037 | 0.805 |
|  | 6 | -0.191 | 0.193 | -0.078 | 0.604 |
